# Genome Evolution in Plants: Complex Thalloid Liverworts (Marchantiopsida)

**DOI:** 10.1093/gbe/evad014

**Published:** 2023-02-02

**Authors:** Anna-Malin Linde, Shilpi Singh, John L Bowman, Magnus Eklund, Nils Cronberg, Ulf Lagercrantz

**Affiliations:** Department of Plant Ecology and Evolution, Evolutionary Biology Centre, Uppsala University, Sweden; School of Biological Sciences, Monash University, Melbourne, Victoria, Australia; School of Biological Sciences, Monash University, Melbourne, Victoria, Australia; Department of Plant Ecology and Evolution, Evolutionary Biology Centre, Uppsala University, Sweden; Biodiversity, Department of Biology, Lund University, Sweden; Department of Plant Ecology and Evolution, Evolutionary Biology Centre, Uppsala University, Sweden

**Keywords:** genome evolution, duplication, transposable elements, sex chromosome evolution, collinearity

## Abstract

Why do some genomes stay small and simple, while others become huge, and why are some genomes more stable? In contrast to angiosperms and gymnosperms, liverworts are characterized by small genomes with low variation in size and conserved chromosome numbers. We quantified genome evolution among five Marchantiophyta (liverworts), measuring gene characteristics, transposable element (TE) landscape, collinearity, and sex chromosome evolution that might explain the small size and limited variability of liverwort genomes. No genome duplications were identified among examined liverworts and levels of duplicated genes are low. Among the liverwort species, *Lunularia cruciata* stands out with a genome size almost twice that of the other liverwort species investigated here, and most of this increased size is due to bursts of Ty3/Gypsy retrotransposons. Intrachromosomal rearrangements between examined liverworts are abundant but occur at a slower rate compared with angiosperms. Most genes on *L. cruciata* scaffolds have their orthologs on homologous *Marchantia polymorpha* chromosomes, indicating a low degree of rearrangements between chromosomes. Still, translocation of a fragment of the female U chromosome to an autosome was predicted from our data, which might explain the uniquely small U chromosome in *L. cruciata*. Low levels of gene duplication, TE activity, and chromosomal rearrangements might contribute to the apparent slow rate of morphological evolution in liverworts.

**Significance**

The rate of genome evolution influenced by, for example, polyploidy, transposable element (TE) accumulation, and chromosomal rearrangements is generally high among plants and in particular within angiosperms. Bryophytes contain smaller and less variable genomes than other nonseed plants, suggesting that the pattern of genome size evolution differs between plant divisions in various aspects. However, the reasons for these observations are lacking as genomic information from bryophyte genomes is limited. Analyses of genome evolution among liverworts revealed a low rate of gene duplication and chromosomal rearrangements and rare bursts of TEs, which can explain the small size and limited variability of liverwort genomes.

## Introduction

Some genomes stay small and simple, while others evolve to become huge (Pellicer and Leitch 2020). Likewise, some genomes show higher levels of stability than others, which might be related to the complexity and diversification of the organisms. High rates of genome size evolution, but not the actual size of the genome, correlate with high rates of speciation in angiosperms, consistent with previous predictions that genome size variability is linked to success in flowering plants (Puttick et al. 2015).

Angiosperm nuclear genomes vary 1,000-fold in DNA content, from around 60 Mb (*Genlisea tuberosa*) to 149,000 Mb (*Paris japonica* [Pellicer et al. 2018]). Whole-genome duplications (WGDs) can account for such variation but also accumulation of transposable elements (TEs; reviewed in Bennetzen and Wang [2014]). In addition, genome size dynamics is influenced by differences in rates of DNA removal, epigenetic silencing of TEs, natural selection, and drift (Tenaillon et al. 2010). Small angiosperm genomes appear to be reduced in size due to effective genome reduction, while genomes of species in the lycophyte genus *Selaginella* appear to be small due to a low rate of genome size increase (Baniaga et al. 2016). Gymnosperm genomes are on average large with low diversity between lineages (16-fold [Pellicer et al. 2018]). These large sizes are likely due to a slow constant accumulation of TEs together with slow removal of DNA (Bennetzen and Wang 2014). Genomes may also evolve through structural changes as a result of translocations, inversions, duplications, and deletions that shuffle the order and orientation of genomic sequences (Bennetzen and Wang 2014). These are often associated with ectopic recombination at repetitive elements and may act as a source of genetic novelty.

Bryophytes occupy a pivotal position in the phylogeny of land plants as the sister group to all other extant land plants. The diversification of bryophytes has been hypothesized to be slow due to an assumed slow rate of morphological change (Crum 1972). It has been speculated that this is accompanied by a slow rate of evolution at the molecular level (Stenøien 2008). Linde et al. (2021) showed that the molecular evolutionary rate, in terms of synonymous substitution rates, was slower compared with angiosperms but not as low as in gymnosperms.

A slow rate of diversification in bryophytes could also be due to a slow rate of structural changes in their genomes. In this study, we focus on liverworts (Marchantiophyta), one of the three major clades of the bryophytes, which are characterized by low variation in genome size and chromosome numbers (Berrie 1960; Bainard et al. 2013), by a lack of known ancient WGD events, and by smaller gene families (Bowman et al. 2017; One Thousand Plant Transcriptomes Initiative 2019). There are currently limited genomic data in bryophytes, with only a few genomes of phylogenetically divergent taxa available; thus, knowledge on the patterns and rates of structural changes is limited (Szövényi et al. 2021).

The aim of this study is to examine and quantify different aspects of structural genome evolution among liverworts. We study taxa representing contrasting divergence times to compare the changes of TE landscape complexity, sequence collinearity, and sex chromosome evolution to identify the processes underlying these structural changes.

## Results

### Low Gene Duplication Rate in Liverworts

It has been estimated that, on average, 65% of the annotated genes in plant genomes have a duplicate copy (Panchy et al. 2016). WGD and tandem duplication account for the majority of plant duplicates in angiosperms, but TE-based mechanisms and retrotransposition also generate a significant number of duplicates (Panchy et al. 2016). Using the same criteria as Panchy et al. (2016), we found that the estimates of duplicated genes among liverworts are similar and in lower range of those of the 45–84% observed in other land plants (table 1; supplementary fig. S1, Supplementary Material online [Panchy et al. 2016]).

**Table 1:** Genomic Features of the Included Species and Subspecies

Abbreviation	MPR	MPM	MPP	LC	MPA	AAN	AAG	KN	PP	SM	PA	AT
Assembly size (Mb)	226	225	223	580	238	119	117	104	472	213^a^	19,600	119
Repeat content (%)^b^	25	29	31	63	40	nd	nd	nd	Nd	nd	nd	nd
No. of annotated genes	19,138	18,806	17,374	16,768	14,478^c^	14,629	25,846	17,055	32,458	22,285	28,354	27,655
No. of duplicated genes	7,312	6,636	6,379	6,901	5,761	6,578	13,602	7,497	16,659	13,718	14,598	18,302
Percent duplicated genes^d^	50	45	45	50	45	54	68	49	76	73	77	79
Total gene length (Mb)	71.9	62.3	56.1	57.1	44.4^c^	28.8	52.6	81.8	107.7	37.9	89.3	65.6
Mean gene length (bp)	3,729	3,313	3227	3,404	3,070	1,972	2,034	4,768	3,271	1,701	3,148	2,375
Mean CDS length (bp)	1,215	1,239	1276	1,269	1,448	1,313	1,206	1,558	1,136	1,145	941	1,217
Mean intron length (bp)	347	373	301	363	322	172	133	499	264	111	1017	159
Mean exon length (bp)	256	287	302	349	241	272	339	294	373	213	312	306
Exons per mRNA ratio	5.2	5.6	5.8	5.3	6.0	4.8	4.5	6.6	5.4	5.6	3	5.3

Note.—MPP, *Marchantia polymorpha ruderalis*; MPM*, Marchantia polymorpha montivagans*; MPP, *Marchantia polymorpha polymorpha*; LC, *Lunularia cruciata*; MPA, *Marchantia paleacea*; AAN, *Anthoceros angustus*; AAG, *Anthorceros agrestis*; KN, *Klebsormidium nitens*; PP, *Physcomitrella patens*; SM, *Selaginella moellendorffii*; PA, *Picea abies*; AT, *Arabidopsis thaliana*. nd, not determined.

Gene statistics for *P. abies* were taken from Nystedt et al. (2013).

a The assembled genome includes two haplotypes of ∼106 Mb (Banks et al. 2011).

b Estimates include microsatellites and low complexity repeats.

c Likely reflecting the lower quality of the annotation, not a reduced number of genes.

d Number of duplicated genes divided by number of annotated genes longer than 150 aa.

In agreement with the low duplication rate, liverwort genomes contain a lower number of genes compared with most other clades of plants (table 1; supplementary fig. S1, Supplementary Material online). The liverwort estimates are within the range of those of the hornworts *Anthoceros angustus* and *Anthorceros agrestis* but lower than that of the lycophyte *Selaginella moellendorffii*. This is not surprising considering the lack of reported ancient WGDs in liverworts and hornworts (Bowman et al. 2017; Lang et al. 2018; Zhang et al. 2020). The average gene size of liverworts is similar to those of other plant clades (table 1). Despite the similar number of genes in *Lunularia cruciata* and *Marchantia polymorpha* (∼17,000 vs. ∼19,000; table 1), the estimated genome size of *L. cruciata* is more than twice that of *M. polymorpha* (table 1).

### The Larger Size of the *L. cruciata* Genome Is Largely Explained by TE Content

**Fig. 1.**
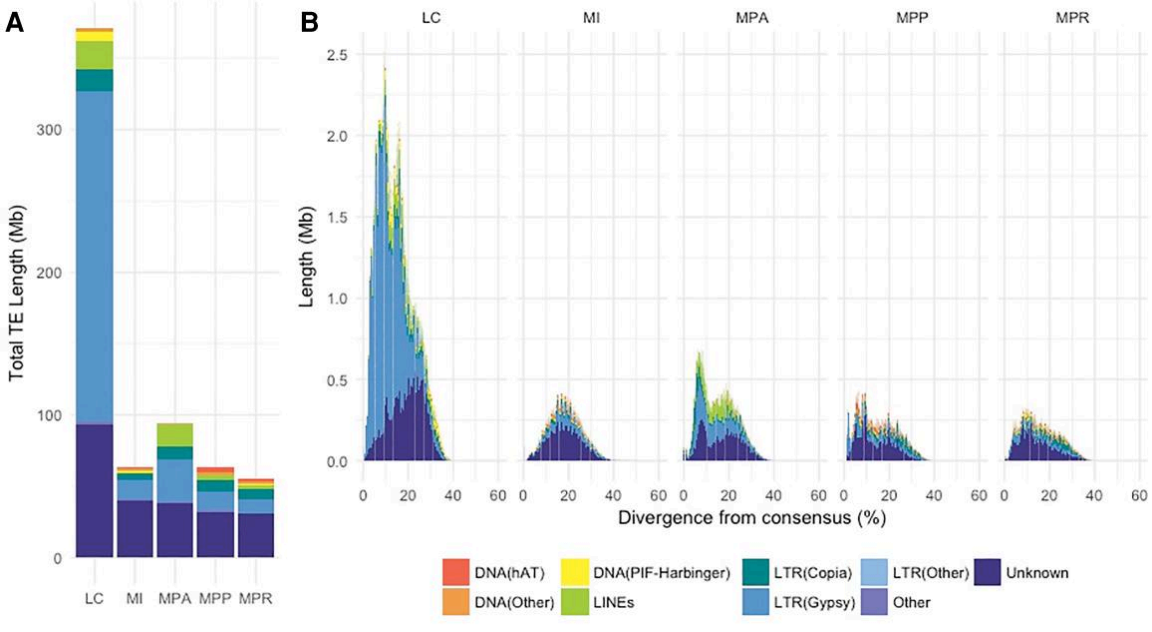
The *Lunularia cruciata* genome is larger due to a high abundance of Ty3/Gypsy retrotransposons. (*A*) Repeat content of the genome assemblies included in this study. (*B*) Abundance (in Mb) of repeats plotted against divergence from consensus (%) in 1% bins. LC, *Lunularia cruciata*; MI, *Marchantia inflexa*; MPA, *Marchantia paleacea*; MPP, *Marchantia polymorpha* subsp. *polymorpha*; MPR, *Marchantia polymorpha* subsp. *ruderalis*.

**Fig. 2.**
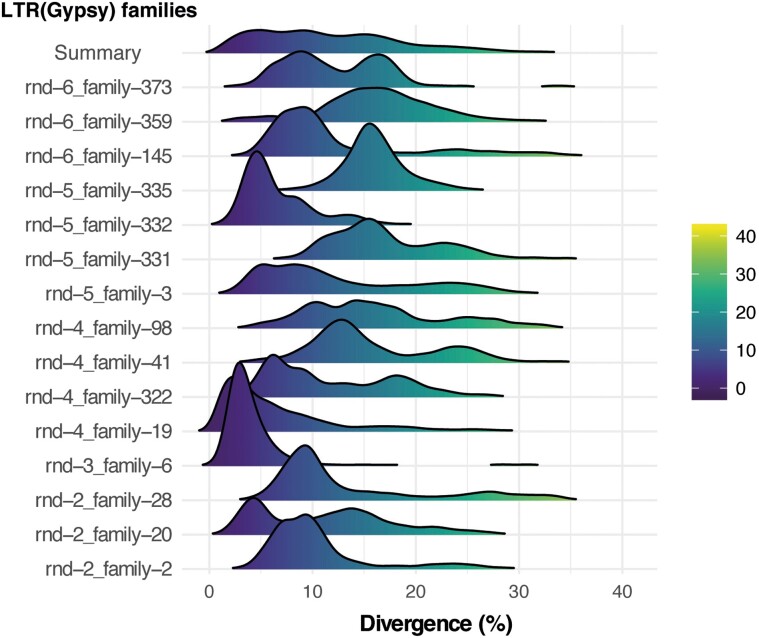
Individual Ty3/Gypsy families in *L. cruciata* were amplified at different time periods. Density plots of divergence from consensus for the 15 largest *L. cruciata* Ty3/Gypsy.

The estimated repeat contents of the liverwort assemblies range from 25% for *M. polymorpha ruderalis* to 63% for *L. cruciata* (table 1). The relatively high repeat content of *L. cruciata* is in agreement with its almost doubled genome size when compared with *M. polymorpha.* To examine the reasons behind this higher repeat content, the landscape of TEs in the liverwort genomes was characterized. Indeed, most of the genome inflation of *L. cruciata* is explained by expansion of TEs (fig. 2*A*, supplementary table S1, Supplementary Material online). The vast majority of this TE expansion emanates from a proliferation of one group of long terminal repeat (LTR) retrotransposons, the Ty3/Gypsy superfamily. The *M. polymorpha ruderalis* genome contains a similar amount of the two main retrotransposons superfamilies Ty1/Copia and Ty3/Gypsy (fig. 1*A* [Montgomery et al. 2020]), while *L. cruciata* contains a large excess of Ty3/Gypsy elements (40% of all repeats). The relatively large proportion of repeats in *L. cruciata* denoted “unknown” may contain liverwort-specific TEs or reflect problems in the assembly of repetitive sequences using short read data.

Bursts of TE transposition can be identified by examining the divergence of individual TEs from the TE family consensus sequence. In *L. cruciata*, more than one peak is obvious in the distribution of divergence time (fig. 1*B*), suggesting more than one burst of proliferation of Ty3/Gypsy elements. Similar but much less pronounced bursts were also evident in the other species that seem to have experienced a slower accumulation of TEs. A more detailed analysis of specific families of Ty3/Gypsy elements in *L. cruciata* reveals a complex pattern of proliferation of specific elements at several time points (fig. 2). This pattern supports the idea that specific elements may mutate to evade hosts’ silencing and proliferate for some time before host silencing reoccurs (Lisch and Slotkin 2011). A comparative analysis of Ty3/Gypsy and Ty1/Copia elements in *L. cruciata* and *M. polymorpha ruderalis* revealed that bursts seem to have occurred at similar time points in the two genomes, but the distributions of divergence are skewed toward lower divergence for *L. cruciata*, especially for Ty3/Gypsy elements (supplementary fig. S2, Supplementary Material online). This suggests either an increased transposon activity in the *L. cruciata* lineage or an increased efficiency of excision of transposed elements in the lineage leading to *M. polymorpha*. More data are needed to determine which of these alternatives are more important, but the latter hypothesis is supported by data on genome size evolution in liverworts (Bainard et al. 2013). The estimated number of full-length LTR retrotransposons, potentially functional, was 2,827 for the male genome of *L. cruciata*—considerably more than the 222 identified full-length LTRs in *M. polymorpha ruderalis* chromosome assembly. This difference supports more recent transposon insertions in *L. cruciata*.

### Slow Rate of Chromosomal Rearrangements in Liverworts

**Fig. 3.**
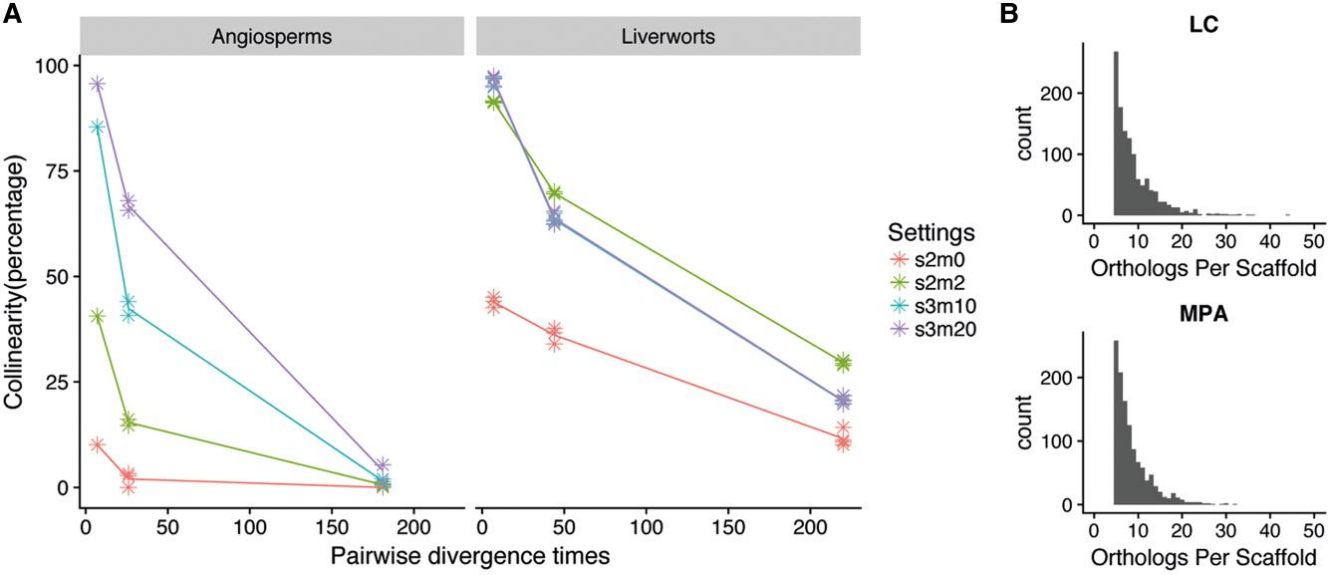
Decrease in collinearity over time is slower in liverworts. (*A*) Percentage of collinear orthologous genes using MCscanX with different settings for *s* (number of genes required to call collinear blocks) and *m* (maximum gaps allowed). One dot represents one pairwise comparison and the lines are drawn through the averaged value for each estimated divergence time. (*B*) Due to the fragmented nature of the assemblies of *L. cruciata* (LC) and *M. paleacea* (MPA), as illustrated here as the distribution of orthologous single-copy genes per scaffold, the most commonly used default values of *s* = 5 and *m* = 25 are not useful for a valid comparison.

**Fig. 4.**
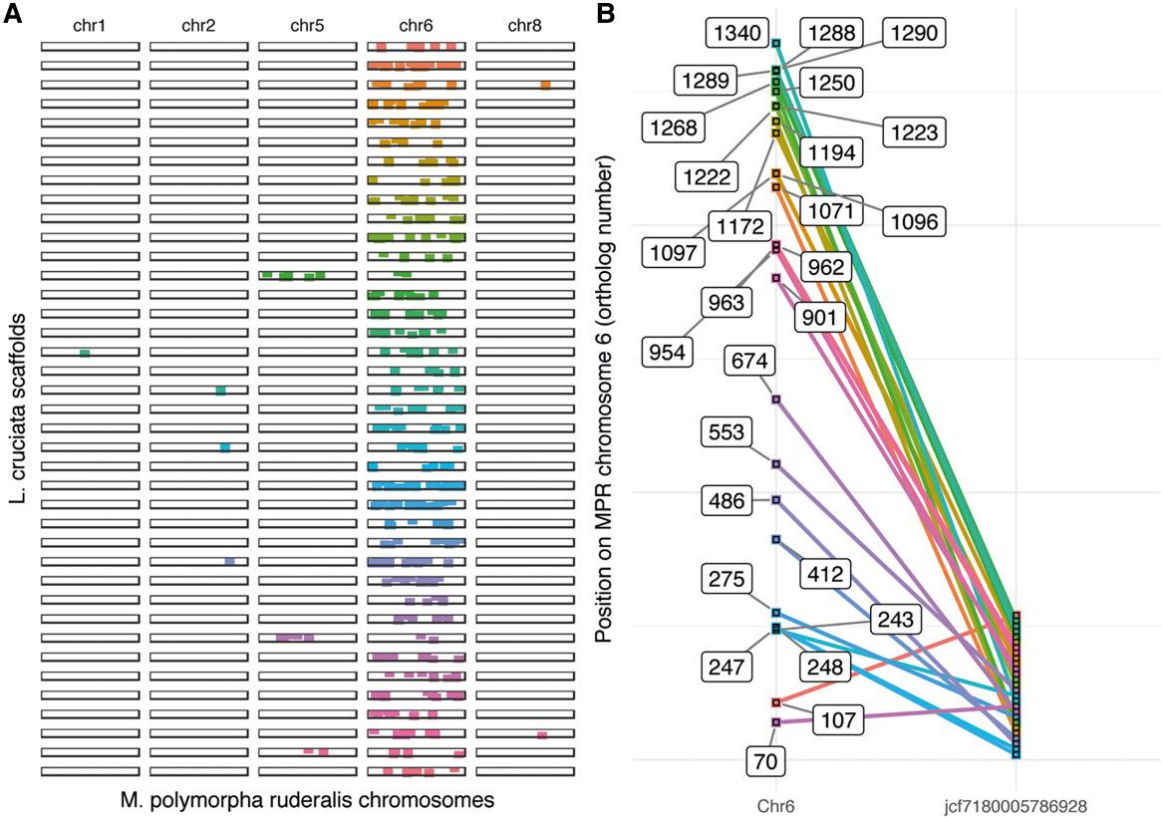
Chromosomal inversions can explain much of the decrease in collinearity between *M. polymorpha* and *L. cruciata*. (*A*) Illustration of rearrangements within and among chromosomes, exemplified by the 36 largest *L. cruciata* scaffolds with at least 10 orthologous hits on *M. polymorpha* subsp. *ruderalis* chromosome 6. Each squared dot represents one gene and all single-copy orthologous genes on the *L. cruciata* scaffolds are printed including the few genes having their orthologous hit on another chromosome than chromosome 6. The scaffolds are color-coded. (*B*) A close-up of the largest *L. cruciata* scaffold (in the number of orthologous single-copy genes) and its collinear relationship with *M. polymorpha* subsp. ruderalis chromosome 6. The genes are numbered after their order of appearance on chromosome 6—gaps in the numbering result from *M. polymorpha* genes that do not have an identified ortholog on this *L. cruciata* scaffold. The lines connecting orthologs have different colors for the sake of clarity.

*Marchantia polymorpha ruderalis* and *L. cruciata* diverged some 220 Ma before the split of the last common ancestor of *Arabidopsis thaliana* and the basal angiosperm *Amborella trichopoda* (181 Ma [Kumar et al. 2017]). A comparison of the decrease of collinearity with divergence times between liverworts and angiosperms indicates a slower rate of chromosomal rearrangements in liverworts than in angiosperms (fig. 3). The assemblies of *L. cruciata* and *Marchantia paleacea* were comparatively more fragmented (fig. 3*B*and*C*). This could potentially bias the collinearity downward. Still, our data including these species suggest a slower decrease in collinearity for liverworts when compared with angiosperms. Within scaffold size limits, most *L. cruciata* single-copy genes have their orthologs on homologous *M. polymorpha* chromosomes, but they are commonly spread over the entire chromosome (fig. 4; supplementary fig. S3, Supplementary Material online).

Thus, our data suggest a multitude of chromosomal rearrangements, including inversions, since the separation of the lineages leading to *L. cruciata* and *M. polymorpha*. It should be noted that the frequency of translocations and other larger-scale-rearrangements (beyond the limited size of the *L. cruciata* scaffolds) is underestimated. Based on available data, it is not possible to estimate and compare rates within the two liverwort lineages. Thus, we cannot conclude if the rate of chromosomal rearrangements is associated with the higher TE activity in *L. cruciata* (Bennetzen and Wang 2014).

### Sex Chromosome Evolution

**Fig. 5.**
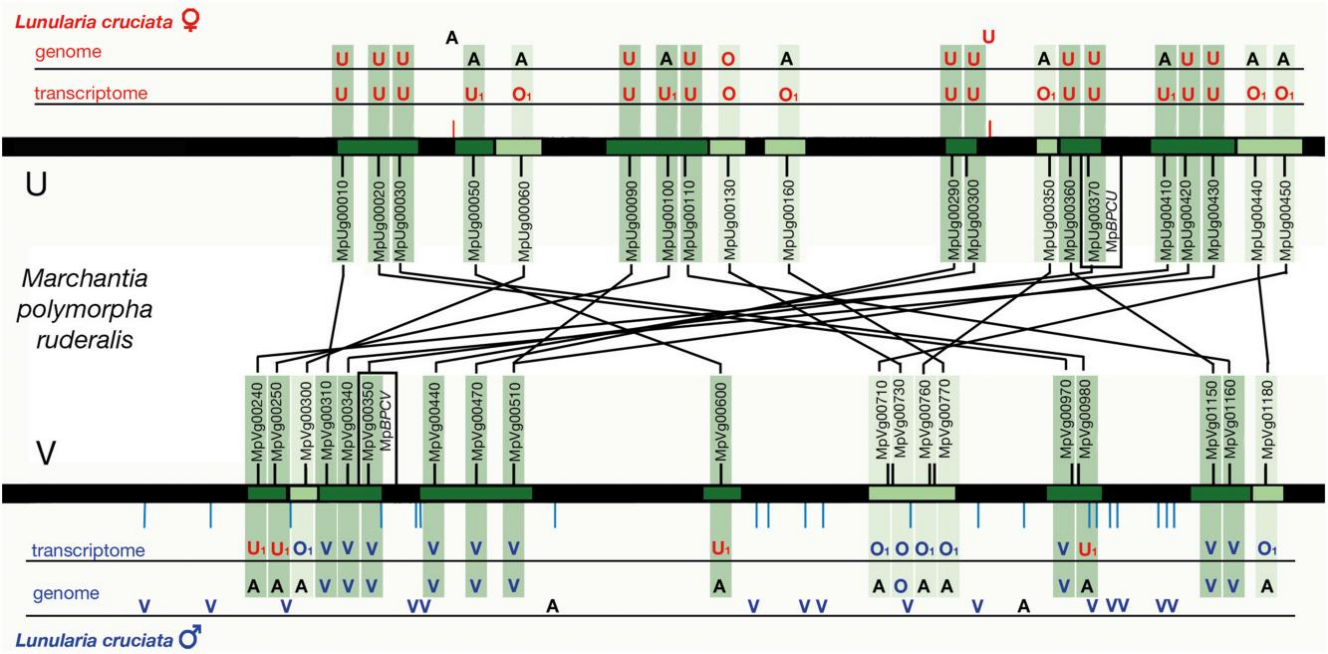
Many *L. cruciata* orthologs of *M. polymorpha* sex chromosome genes are also sex-specific. The *M. polymorpha* U and V chromosome schematics are central (Bowman et al. 2017; Montgomery et al. 2020; Iwasaki et al. 2021). Gametologs are listed between the U and V chromosomes, with phylogenetically conserved U- and V-specific genes represented by short outward pointing lines (supplementary table S4, Supplementary Material online). Gametolog pairs predicted to have been in nonrecombining regions, presumably on sex chromosomes of the common ancestors of *Lunularia* and *Marchantia*, are in dark green, whereas those predicted to have been in recombining regions (either pseudoautosomal or autosomal) in the common ancestor are in light green (Iwasaki et al. 2021). The results of phylogenetic analyses of *L. cruciata* orthologs of *M. polymorpha* gametologs (supplementary fig. S4 and table S4, Supplementary Material online) are presented for the male and female transcriptomes and genomes. Orthologs are classified as U or V if the detected ortholog is more closely related to the U or V *M. polymorpha* gametolog, respectively, than the *L. cruciata* sequences to each other. Orthologs are defined as O (outgroup) if the two *M. polymorpha* gametologs are more closely related to each other than the *L. cruciata* ortholog. If the male and female transcriptomes possess the same gametolog, it is demarcated with a “1.” For the genome, if the gene sequence is specific to the female or male genome, it is demarcated in red (U or O) or blue (V or O), respectively. If the sequence is found in both female and male genomes, it is demarcated in black (*A*, autosomal or pseudoautosomal). In each case where a single sequence is found in the transcriptomes, the gene is predicted to be (pseudo)autosomal. Note that although many of the *L. cruciata* genes represented are predicted to reside on the sex chromosomes, the chromosomal order and orientation of the genes in *L. cruciata* is unknown.

*Lunularia cruciata* is dioicous, that is having separate male and female haploid individuals, with the presence of U or V sex chromosomes, respectively, and this is the presumed ancestral condition in liverworts (Berrie 1960; Bowman 2016; Iwasaki et al. 2021). In nonrecombining regions of the sex chromosomes, U and V chromosomes possess divergent gametolog pairs descended from a common ancestral autosomal gene. Previously, *L. cruciata* orthologs of *M. polymorpha* gametologs were identified from two transcriptomes representing female and male individuals (Iwasaki et al. 2021; supplementary table S3, Supplementary Material online). Many orthologs from the female transcriptome are descended from a presumed U-linked gene in the common ancestor of *L. cruciata* and *M. polymorpha*, with some others having an outgroup phylogenetic relationship with the two *M. polymorpha* gametologs (fig. 5; supplementary fig. S4, Supplementary Material online; supplementary table S3, Supplementary Material online). In contrast, whereas some male transcriptome orthologs descended from a presumed V-linked gene in the common ancestor or have an outgroup relationship, others are identical to the female transcriptome genes presumed descended from ancestral U-linked genes (fig. 5; supplementary fig. S4, Supplementary Material online; supplementary table S3, Supplementary Material online).

Although the structure of *L. cruciata* sex chromosomes is presently unresolved, genes residing on the U or V chromosomes should be present only in the female or male genomes, respectively. We first confirmed that in each case the gametolog orthologs of the female and male genomes matched those of the female and male transcriptomes, respectively (fig. 5; supplementary fig. S4, Supplementary Material online; supplementary table S3, Supplementary Material online). We next examined whether the gametolog orthologs were unique to the female or male genomes, suggesting that the sequences reside on the U or V chromosome, or whether they were shared, which would suggest that sequences are autosomal (or pseudoautosomal). In each case where gametolog ortholog pairs were identified with distinct sex-specific sequences, they are located on genomic scaffolds that are unique to either the female or male. In contrast, in every case where the male and female gametolog orthologs have similar or identical sequences, the genomic scaffolds on which these genes reside are found in both the female and male genomes. Lastly, at least some orthologs of phylogenetically conserved *M. polymorpha* U- and V-specific genes may also be U- or V-linked in *L. cruciata* as they reside on scaffolds containing sequences found in genomic assemblies derived from only one sex (fig. 5; supplementary table S4, Supplementary Material online). This is in contrast to scaffolds presumed to be autosomal where corresponding scaffolds in the opposite sex are typically >99% identical.

## Discussion

It has been hypothesized that bryophytes, being structurally simple organisms with a predominantly haploid life cycle, have experienced slow molecular evolution, similar to their seemingly slow morphological diversification. Linde et al. (2021) showed that the silent site substitution rates of bryophytes are not lower when compared with vascular plants as a group. Bryophyte rates were lower than for angiosperms but higher than gymnosperms; thus, low substitution rates cannot alone explain a difference in morphologic evolution.

Bryophytes are characterized by small genomes. The mean (median in parentheses) of bryophyte genome sizes has been estimated to be 244 Mb (205 Mb), 1,844 Mb (751 Mb), and 504 Mb (433 Mb) for hornworts, liverworts, and mosses, respectively (Pellicer et al. 2018). Liverworts are subdivided into three classes: the species-rich classes Marchantiopsida, Jungermanniopsida, and the smaller class Haplomitriopsida. Species belonging to Marchantiopsida show limited genome sizes (∼5-fold variation [Pellicer and Leitch 2020]) compared with Jungermanniopsida (∼100-fold variation [Pellicer and Leitch 2020]). This variation is very limited compared with the angiosperm lineage, where genome sizes vary 2,440-fold with a mean (median in parentheses) of 5,020 Mb (1,663 Mb), and although some of the largest genomes are observed within the angiosperms, their genome sizes are skewed toward smaller genomes (Pellicer et al. 2018).

### Low Levels of Gene Duplication in Marchantiophyta

A low degree of gene duplication has been reported for *M. polymorpha ruderalis* (Bowman et al. 2017). Our data now suggest that the percentage of duplicated genes and tandem duplicated is similar between all the studied Marchantiopsida liverworts despite a large phylogenetic distance in the case of *L. cruciata* and *M. polymorpha ruderalis*. The low number of duplicated genes in Marchantiophyta liverworts can to a large extent be explained by a lack of ancient WGD events (Bowman et al. 2017). A considerable number of paralogs were all the same detected in liverworts, probably to a large extent resulting from tandem or segmental duplications (fig. 1). Still, tandem duplications in *Marchantiophyta* are at the lower range compared with angiosperms (Bowman et al. 2017). Gene number estimates within Marchantiophyta are between those of the hornwort *A. angustus* and *A. agrestis* and similar to those of the charophyte alga *Klebsormidium nitens*. The lower gene number estimate in *A. angustus* is consistent with a reported high rate of gene family loss in this species (Zhang et al. 2020). As gene duplication may increase evolutionary potential (Ohno 2013), the low gene numbers of Marchantiophyta species are consistent with a reduced evolvability of the group.

### Bursts of Transposable Element Accumulation in *L. cruciata*

Despite a limited variation in genome size among representatives of Marchantiopsida, the genome of *L. cruciata* is approximately twice the size when compared with that of *M. polymorpha.* This difference is mainly due to a higher copy number of a few families of TEs in *L. cruciata* that are the result of several bursts of amplification. These results show that such bursts, that are common in angiosperms, also occur in liverworts. However, extrapolating from our results this kind of burst seems rare, at least among Marchantiopsida. Even though the TE content is generally lower in the studied liverworts when compared with other most plant species, a similar collection of elements was observed. The most common TE elements were Ty3/Gypsy and Ty1/Copia, as has been reported in other plants (Voytas et al. 1992; Suoniemi et al. 1998). The ratio of Ty3/Gypsy to Ty1/Copia elements varies in the studied liverwort species, with Ty3/Gypsy being the most abundant in *L. cruciata*, whereas the two are present in similar proportions in *M. paleacea.* Most of this variation can be explained by multiple bursts of Gypsy families in *L. cruciata*.

The larger transposon accumulation over time in *Lunularia* when compared with the *Marchantia* species is not associated with any known radiation, and the order Lunulariales is considered monotypic with a single species, *L. cruciata*, in contrast to the much more speciose Marchantiales (Söderström et al. 2016).

### Low Rate of Chromosomal Rearrangements in Marchantiopsida

Overall, there is a much higher average microsynteny across placental mammals compared with angiosperms (Zhao and Schranz 2019). Wang et al. (2012) reported 0.6–6.7% collinear ortholog pairs in pairwise comparisons between monocot and eudicot species that diverged approximately 160 Ma (Kumar et al. 2017). Because different methods and thresholds have been used, those values are not directly comparable with the values obtained in the present study. Still, regardless of the settings used in our analysis, the percentage collinear gene pairs between Marchantiopsida liverwort species pairs is always higher than those obtained from angiosperm pairs. Thus, a relatively low rate of chromosomal rearrangements is suggested for species within Marchantiopsida. Although the fragmented assembly of *L. cruciata* precludes a direct comparison of rates of inversions versus translocations, our results are consistent with the conclusion that structural changes within plant chromosomes are far more abundant than those between chromosomes (Dvorak et al. 2018). Linde et al. (2020) found that chromosome 2 of *M. polymorpha* subsp. *montivagans* was distinctly more divergent than any other parts of the genome in pairwise comparisons with the other subspecies of *M. polymorpha.* A higher degree of structural rearrangements on chromosome 2 of subsp. *montivagans* suggested that it might have been more strongly protected against introgression than other parts of the genome. This chromosome also had a higher proportion of transposons.

### Sex Chromosome Evolution in *L. cruciata*

Among liverworts, the karyotype of *L. cruciata* with the V chromosome larger than the U is unusual and in contrast to the vast majority of liverworts with dimorphic sex chromosomes in which the U is larger than the V (Lorbeer 1934; Sergio and Viana 1973). A translocation of a fragment of the U chromosome to an autosomal location could account for this unique karyotype. The pattern of *L. cruciata* gametolog evolution is consistent with the hypothesis that a segment composed of both previously nonrecombining regions (including three gametologs and one U-specific gene) and potentially ancestrally pseudoautosomal regions was translocated from the U to an autosome. Consistent with this hypothesis and as predicted by Muller's ratchet (Muller 1932), the corresponding V-gametologs appear to have been lost.

## Conclusions

Our analyses reveal clear differences in the patterns of genome evolution between liverworts and angiosperms resulting in smaller and less variable liverwort genomes. First, although angiosperms have gone through several rounds of ancient WGDs, no ancient WGD has been identified for liverworts. Even though tandem duplications are not uncommon in liverworts, their genomes generally contain fewer genes. This difference could restrict the evolutionary potential for liverworts, as less new genetic material is available for mutation, drift, and selection to act upon. Second, the rate of structural changes in terms of both genome size and rearrangements seems low when compared with angiosperms. These differences might be the result of lower transposon activity. We did observe bursts of primarily LTR TEs in liverworts, similar to what is prevalent in angiosperms. However, such events seem to be much more rare in liverworts. As transposon activity is thought to provide additional mutations that might speed up evolution in terms of both rates of adaptation and speciation (Chénais et al. 2012; Rebollo et al. 2012), the association between a low TE activity and a low rate of evolutionary change in liverworts is not unexpected.

## Materials and Methods

### Genome Assembly and Annotation

Genomes of five *Marchantiopsida* liverwort species/subspecies were included: *M. polymorpha* subsp. *ruderalis*, *M. polymorpha* subsp. *polymorpha*, *M. polymorpha* subsp. *montivagans*, *Marchantia inflexa*, and *L. cruciata.* Assembly and annotation of *M. polymorpha* subsp. *ruderalis* are publicly available (Bowman et al. 2017; Montgomery et al. 2020; Iwasaki et al. 2021) and so is the assembly of *M. inflexa* (Marks et al. 2019), which was used for only repeat characterization. Sequencing, assembly, and annotation of *M. polymorpha* subsp. *polymorpha* and subsp. *montivagans* are described in Linde et al. (2020). The genome of *M. paleacea* subsp. *diptera* was sequenced and assembled as described in Radhakrishnan (2017) and summarized in Linde et al. (2020).

The female genome of *L. cruciata* is described in Linde et al. (2021). BUSCO (Simão et al. 2015), the eukaryote_odb9 lineage data set, and CEGMA were used to assess the completeness of the assembly, which was 87.4% (89.1%) according to BUSCO and 90.32% (95.56%) according to CEGMA, with the rates in parenthesis also including fragmented/partial hits. GenomeScope (http://qb.cshl.edu/genomescope/) was used to verify the estimates for genome size and repeat content using a kmer-based statistical approach on the raw reads giving an estimate of the genome size (581 Mb) similar to the assembly size (580 Mb) but a lower estimate of the repeat content (35%) compared with the value estimated as described below in the repeat annotation section, likely due to the failure to detect older and more divergent repeats. Annotation of *L. cruciata* was performed as described in Linde et al. (2020). BUSCO was used to assess the completeness of the annotations. The *M. polymorpha ruderalis* annotation version 3.1, which is considered to be more or less complete, had a completeness of 97.4 (97.7%). The completeness of the annotations of *M. polymorpha* subsp. *montivagans*, subsp. *polymorpha*, *L. cruciata*, and *M. paleacea* was 92.8% (97.8%), 86.8% (96.7%), 85.8 (95.4%), and 90.8% (96.7%), respectively (fragmented hits included in numbers within parenthesis).

The genome of a male *L. cruciata* was assembled using the data set SRR8246012 (Dong et al. 2019). The assembly was performed using MaSuRCA version 3.4.2 (Zimin et al. 2013), with pair end, using default settings. The genome size of male *L. cruciata* was estimated on the basis of size of the female genome (581 Mb) and a 519 Mb genome was assembled. BUSCO (Simão et al. 2015), together with the eukaryota data set, was used to assess the completeness of the assembly, which was 93.4% (97.7%), with the rate in parenthesis including not only the complete but also the fragmented/partial hits. Assembly statistics are given in supplementary table S2, Supplementary Material online. Both *L. cruciata* assemblies with annotations are available at genomevolution.org (IDs 64629, 64630, and 64631).

### Sex Chromosome Phylogenetics

Predicted *L. cruciata* gametolog orthologs were identified from available genome and transcriptome sequence data (Dong et al. 2019; One Thousand Plant Transcriptomes Initiative 2019; Iwasaki et al. 2021). Nucleotide sequences were manually aligned as amino acid translations using Se-Al v2.0a11 (http://tree.bio.ed.ac.uk/software/seal/). Ambiguously aligned sequences were removed and alignments of nucleotides were employed in subsequent Bayesian phylogenetic analysis using MrBayes 3.2.1 (Huelsenbeck and Ronquist 2001; Huelsenbeck et al. 2001). The Bayesian analysis for the nucleotide data set was run for 500,000 generations, which was sufficient for convergence of the two simultaneous runs (split frequencies < 0.05). To allow for the burn-in phase, the initial 50% of the total number of saved trees was discarded. The graphic representation of the trees was generated using the FigTree (version 1.4.0) software (http://tree.bio.ed.ac.uk/software/figtree/). Nucleotide alignments are provided in supplementary file S1, Supplementary Material online.

## Supplementary Material

evad014_Supplementary_Data

## Data Availability

All sequence data analyzed in this research were previously published as described above. Genome assemblies and annotations for *L. cruciata* were produced based on published data and are available at genomevolution.org (IDs 64629, 64630, and 64631).
