# Supplementary material for: Genome Evolution in Plants: Complex Thalloid Liverworts (Marchantiopsida)
Source: Genome Biol Evol. 2023 Feb 2;15(3):evad014. doi: 10.1093/gbe/evad014 (PMC9985172; doi:10.1093/gbe/evad014)
Supplement: evad014_Supplementary_Data [file evad014_supplementary_data.zip › Supplemental Data_Linde_et_al_21dec.docx]

#### Title: Genome evolution in liverworts

A**uthors:** Anna-Malin Linde^1^, Shilpi Singh^2^, John L. Bowman^2^, Magnus Eklund^1^, Nils Cronberg^3^, Ulf Lagercrantz^1^

**Journal:**

**Doi:**

^1^ Department of Plant Ecology and Evolution, Evolutionary Biology Centre, Uppsala University, Norbyvägen 18D, SE-75236 Uppsala, Sweden.

^2^ School of Biological Sciences, Monash University, Melbourne, Victoria 3800, Australia.

^3^ Biodiversity, Department of Biology, Lund University, Ecology Building, SE-223 62 Lund, Sweden.

**Contents**

*Supplemental methods* 2

#### Gene comparisons 2

Phylogeny 2

Repeat annotation 2

Detection of duplicated genes 3

Detection of synteny and collinearity 3

*Supplemental Tables* 4

Table S1 Repeat content in liverwort genomes 4

Table S2 Assembly statistics, Lunularia male genome 4

Table S3 “Lunularia sex chromosome analysis” Excel file

Table S4 “Lunularia sex chromosome analysis” Excel file

Table S5 Single copy orthologs for collinearity analysis 5

*Supplemental Figures*

Figure S1 5

Figure S2 6

Figure S3 7

Figure S4 8

*Supplemental File*

File S1 Alignments used for phylogenetic trees presented in Figure S4

*References* 11

**Supplemental Methods**

#### Gene comparisons

Gene model annotations of additional plant genome assemblies representing different plant lineages were included for comparison. These were the charophyte algae *Klebsormidium nitens* (KN), the hornworts *Anthoceros angustus* (AAN) and *A. agrestis* (AAG), the moss *Physcomitrium patens* (PP), the non-seed vascular plant *Selaginella moellendorffii* (SM), the angiosperm *Arabidopsis thaliana* (AT) and the gymnosperm *Picea abies* (PA). Annotation and sequence files were downloaded from the following sources: KN (V 1.1) http://www.plantmorphogenesis.bio.titech.ac.jp/~algae_genome_project/klebsormidium/; AAN 10.5061/dryad.msbcc2ftv; AAG https://www.hornworts.uzh.ch/en/download.html;

AT (Araport11) https://www.arabidopsis.org/; PP (V3.3), and SM (v.1.0) https://phytozome-next.jgi.doe.gov; and PA (V1.0 only high confidence (HC) genes) http://congenie.org.

The script *gff3_sp_statistics.pl* from the NBIS annotation toolkit (https://github.com/NBISweden/GAAS/) was used to calculate the gene statistics with the annotation GFF3-files as input. Comparisons are based on the longest isoform of each gene.

#### Phylogeny

The phylogenetic relationship was taken from timetree.org[1], where a list of species was uploaded and the resulting phylogeny based on summarized estimated divergence times were exported as *newick*. The species not included in the database were added manually[2][3]. The phylogeny was visualized using the plotTree.barplot function in the R package Phytools[4].

#### Repeat annotation

RepeatModeler version 1.0.8_RM4.0.7 (Smit, AFA, Hubley, R. *RepeatModeler Open-1.0*.
2008-2015 <http://www.repeatmasker.org>), was used for *de novo*  repeat family identification, and this species-specific repeat-library was used with RepeatMasker version 4.0.7 (Smit, AFA, Hubley, R & Green, P. *RepeatMasker Open-4.0*. 2013-2015 http://www.repeatmasker.org) to characterize the TE landscape. LTRharvest[5] and LTRdigest together with the GyBD collection[6] and plant RNA database (<http://plantrna.ibmp.cnrs.fr/>) was used to find full-length and potentially functional LTRs in the genome assemblies.

#### Detection of duplicated genes

The criteria to define paralogs were taken from Panchy et al. (2016) to make it comparable to the values reported in that study. A gene was considered duplicated if it was significantly similar to another gene in a BLAST search using the criteria identity ≥ 30%, aligned region ≥ 150 amino acids and e-value ≤ 10^-5^,[7]. The percentage of duplicated genes was obtained by dividing with the number of annotated genes larger than 150 amino acids. The number of tandem duplicated genes was obtained using *duplicate gene classifier* with default settings in the MCscanX toolkit[8].

#### Detection of synteny and collinearity.

Single-copy orthologs for the liverwort species and angiosperm species presented in Table S5 were extracted using Orthovenn2[9]. MCscanX_h[8]was used to estimate the percentage of collinear genes for each pairwise comparison. Different combination of s (number of genes required to call collinear blocks) and m (maximum gaps allowed) were tested. The gff3-files were filtered to only include scaffolds with five or more located orthologs. The number of ortholog pairs for each pairwise comparison is reported in **Table S5**. The angiosperm gff3-files were downloaded from the Ensembl Plant database (release January 2019) to get gene names consistent with the single-copy proteins downloaded from orthovenn2.

The single-copy orthologous pairs for *L. cruciata* and *M. polymorpha ruderalis* were used to illustrate the pairwise collinearity. To visualize the actual locations of the orthologs on scaffolds and chromosomes, 36 of the largest *L. cruciata* scaffolds having at least 10 orthologous hits on *M. polymorpha subsp. ruderalis* chromosome 6, were extracted and their positional relationships were plotted using the R package ggplot. In addition, the largest (in number of orthologous single-copy genes) *L. cruciata* scaffold (jcf7180005786928) was used to visualize, in more detail, the collinearity between the orthologs on this scaffold and *M. polymorpha subsp. ruderalis* chromosome 6.

**Supplemental Tables**

**Table S1**. Percentage of different repeat types in liverwort genome sequences.

| Repeat type | **MPR** | **MPM** | **MPP** | **LC** | **MPA** |
| --- | --- | --- | --- | --- | --- |
| SINEs | 0.0 | 0.3 | 0.1 | 0.0 | 0.0 |
| LINEs | 1.0 | 1.3 | 1.4 | 3.3 | 6.2 |
| LTR elements | 7.7 | 10.6 | 10.5 | 42.2 | 16.4 |
| DNA elements | 1.7 | 1.8 | 2.4 | 1.5 | 0.4 |
| Unclassified | 13.1 | 11.5 | 14.6 | 15.5 | 15.7 |
| Total interspersed | 23.6 | 25.6 | 29.0 | 62.6 | 38.6 |
| Satellites | 0.0 | 0.0 | 0.0 | 0.1 | 0.0 |
| Simple repeats | 1.0 | 3.3 | 0.9 | 0.3 | 1.0 |
| Low complexity | 0.2 | 0.2 | 0.2 | 0.1 | 0.2 |
| Total | 24.8 | *29.1* | 31.1 | 63.1 | 39.8 |

Abbreviations: *M. polymorpha* subsp. *ruderalis* (MPR), *M. polymorpha polymorpha* (MPP), *M. polymorpha* subsp. *montivagans* (MPM), *Marchantia paleacea* subsp. *dipera* (MPA), *L. cruciata* (LC).

**Table S2.** Assembly statistics and completeness of *Lunularia cruciata* male genome assembly.

| **Statistics** | ***L.cruciata* Male** |
| --- | --- |
| # contigs (>=1000 bp) | 20,098 |
| Total length (>=1000 bp) | 514,694,558 |
| Largest contig size | 458,794 |
| N50 | 57,015 |
| L50 | 2,732 |
| Assembly completeness (BUSCO), Completed (Complete + Fragmented) | 93.4% (7.7%) |
| Annotated gene models | 23,502 |

Table S5. Total number of single-copy orthologous gene pairs (above diagonal), and estimated pairwise divergence times (MY, below diagonal).

| Liverworts | |  |  |  |  | Angiosperms | | | | |
| --- | --- | --- | --- | --- | --- | --- | --- | --- | --- | --- |
|  | **MPR** | **MPP** | **MPM** | **MPA** | **LC** |  | **AT** | **AL** | **BR** | **AM** |
| MPR |  | 9446 | 9547 | 6961 | 5855 | **AT** |  | 5868 | 5868 | 5836 |
| MPP | 7 |  | 9523 | 6960 | 5857 | **AL** | 7 |  | 5869 | 5837 |
| MPM | 7 | 7 |  | 7019 | 5901 | **BR** | 26 | 26 |  | 5837 |
| MPA | 44 | 44 | 44 |  | 4384 | **AM** | 181 | 181 | 181 |  |
| LC | 220 | 220 | 220 | 220 |  |  |  |  |  |  |

Abbreviations: *M. polymorpha* subsp. *ruderalis* (MPR), *M. polymorpha polymorpha* (MPP), *M. polymorpha* subsp. *montivagans* (MPM), *Marchantia paleacea* subsp. *dipera* (MPA), *L. cruciata* (LC), *A. thaliana* (AT), *A. lyrata* (AL), *B. rapa* (BR), *A. trichopoda* (AM).

**Supplemental Figures**

**Figure S1***.* Estimated number of genes and number of duplicated genes in selected plant species. On the color scale from light gray to dark gray are total number of genes in the annotation (all genes), the number of genes ≥ 150 amino acids (w/o short genes), the number of duplicated genes ≥ 150 amino acids (Duplicated) and the number of tandem duplicated genes (Tandem). The phylogenetic relationship of the species compared is also shown with branch lengths correspond to the divergence in millions of years,

**Figure S2.** Density plots of divergence from consensus for Ty3/Gypsy and Ty1/Copia elements in *M. polymorpha ruderalis* and *L. cruciata*. Elements in families with at least 1000 bp were included.

**
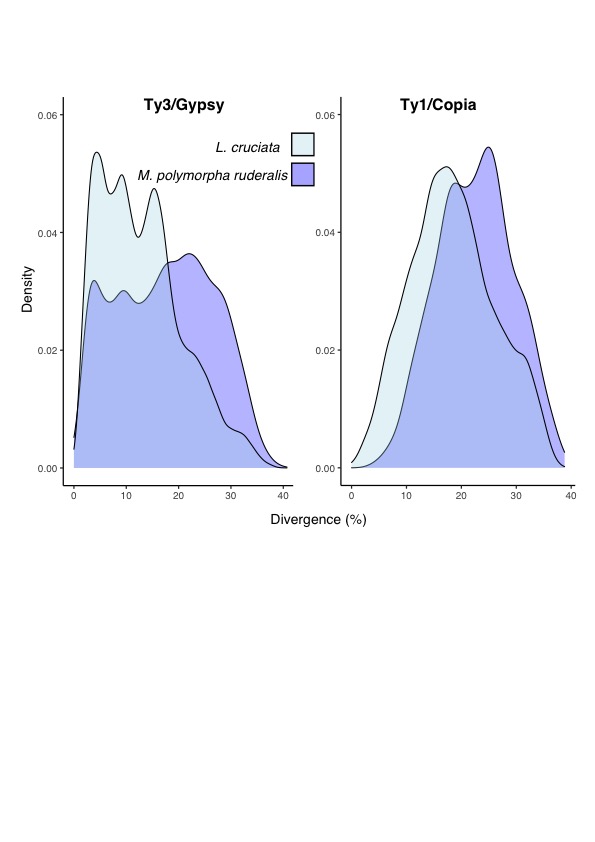
**

**Figure S3.** The syntenic relationship between *M. polymorpha ruderalis* chromosomes and 125 *L. cruciata* scaffolds. Only scaffolds with 10 or more single-copy orthologs shown. The position on y-axis reflects the scaffold number, sorted by its orthologs position in *M. polymorpha ruderalis* genome. The position on x-axis reflects the physical position on the chromosome of the ortholog. Color coded based on which chromosome the *M. polymorpha* ortholog in a pair is located. Each squared dot represents one gene pair (or more, as they might not be distinguishable if located in close proximity).

Figure S4. Phylogenetic trees of the 19 *M. polymorpha* gametolog pairs

Phylograms of the *M. polymorpha* gametolog pairs reconstructed using nucleotide alignments. *M. polymorpha* gene names at top of each phylogram represent the U gametolog. Taxa are color-coded as follows: Haplomitriopsida, black; Marchantiopsida, purple; Jungermanniopsida, red. Clades in which the *Marchantia* *polymorpha* U-chromosome genes reside are highlighted in yellow, and clades in which the *Marchantia* *polymorpha* V-chromosome genes reside are highlighted in Blue. *Lunularia cruciata* sequences included are from a female transcriptome (TXVB nomenclature[11] and marked as
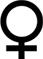
) and male transcriptome (DN nomenclature[12] and marked as
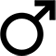
); and a female genome (jcf nomenclature; this study). The male genome sequences were not included as they are identical to the male transcriptome sequences. Numbers at branches indicate posterior probability values. Trees are rooted based on previous phylogenetic information[10]. Nucleotide alignments are presented in Supplemental File S1.


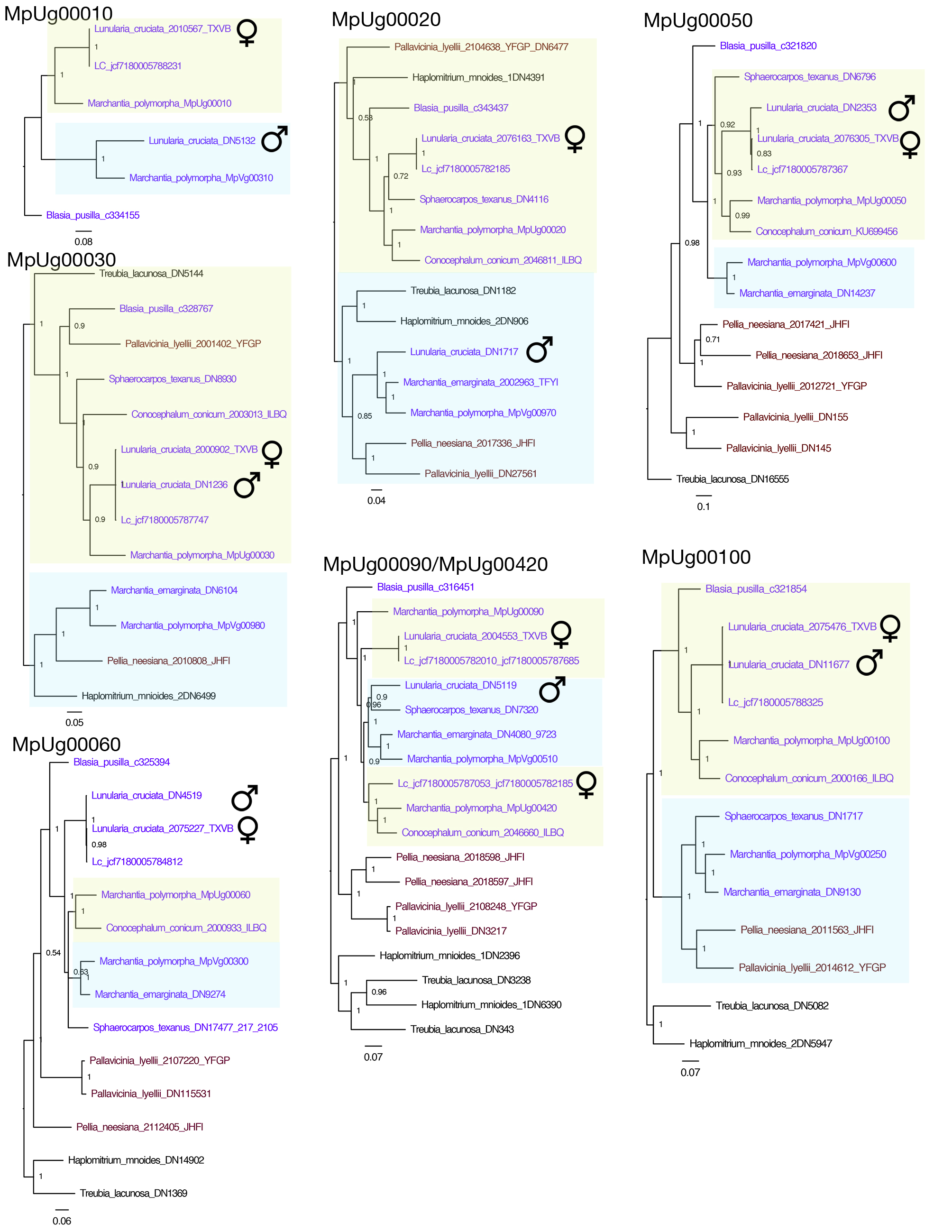


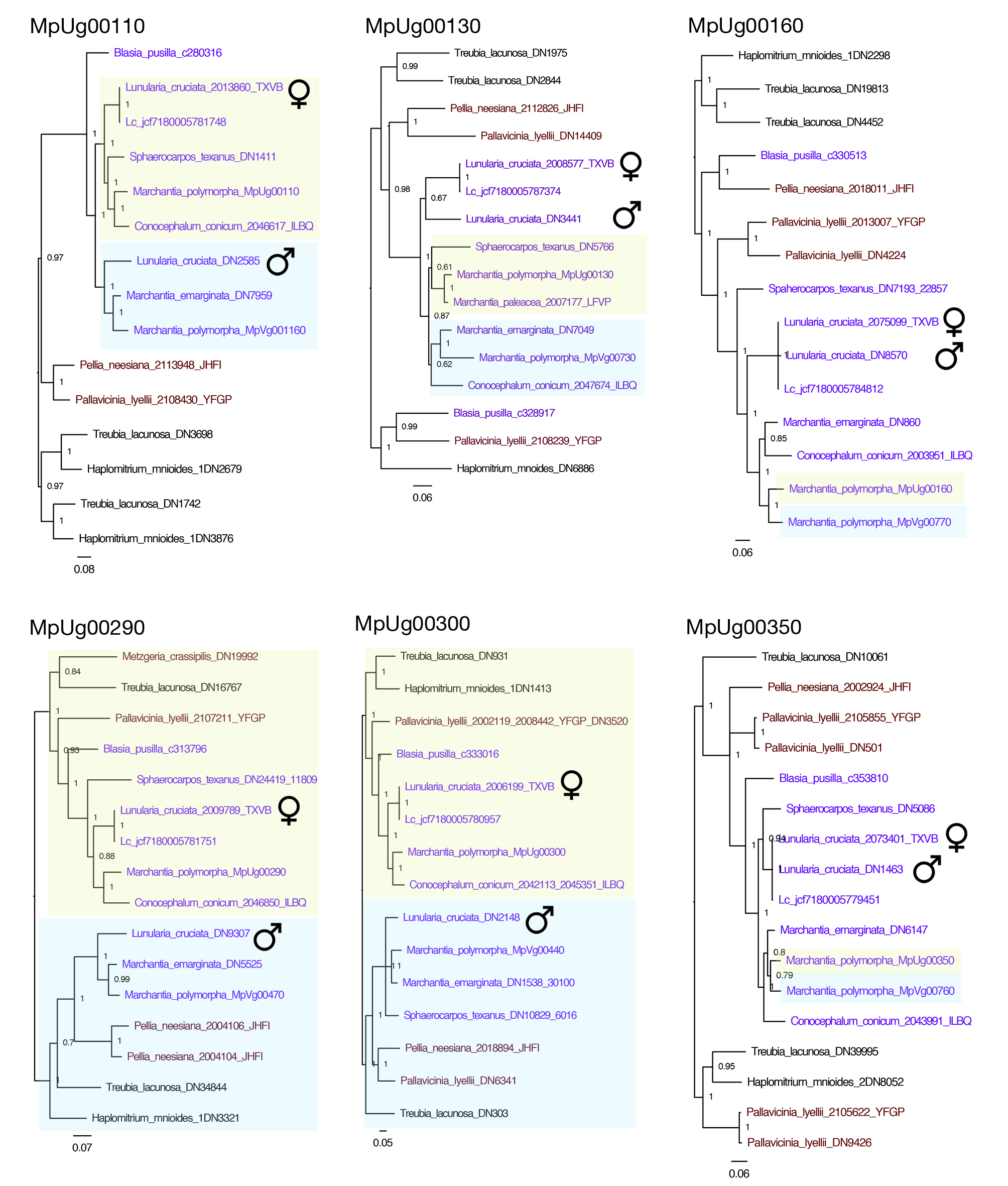


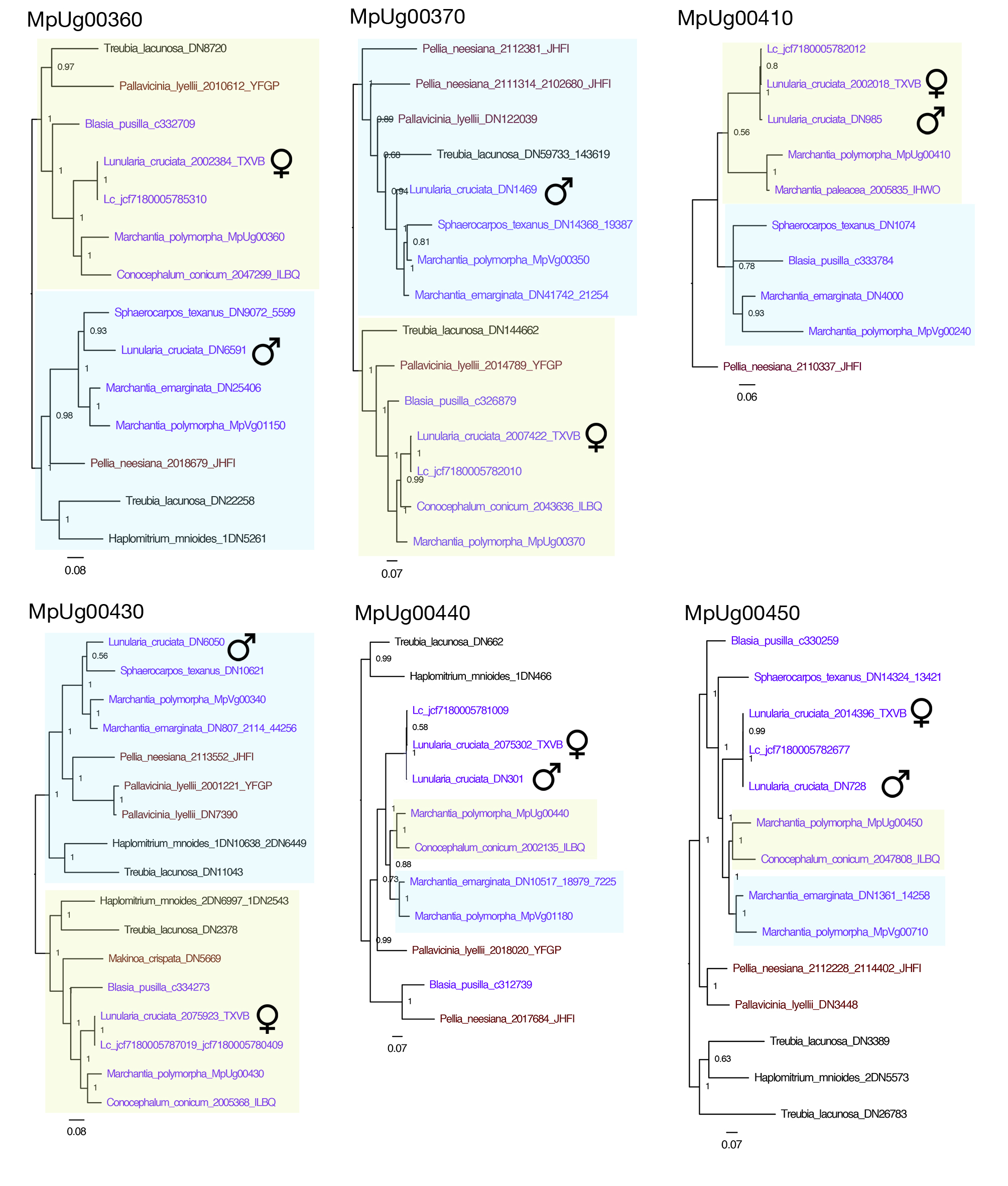


References

1. Kumar S, Stecher G, Suleski M, Hedges SB. 2017 TimeTree: A Resource for Timelines, Timetrees, and Divergence Times. *Mol Biol Evol* 34, 1812–1819. (doi:10.1093/molbev/msx116)

2. Marks RA, Smith JJ, Cronk Q, Grassa CJ, McLetchie DN. 2019 Genome of the tropical plant Marchantia inflexa: implications for sex chromosome evolution and dehydration tolerance. *Sci Rep* 9, 8722. (doi:10.1038/s41598-019-45039-9)

3. Villarreal A JC, Crandall-Stotler BJ, Hart ML, Long DG, Forrest LL. 2016 Divergence times and the evolution of morphological complexity in an early land plant lineage (Marchantiopsida) with a slow molecular rate. *New Phytol.* 209, 1734–1746. (doi:10.1111/nph.13716)

4. Revell LJ. 2012 phytools: an R package for phylogenetic comparative biology (and other things). *Methods in Ecology and Evolution* 3, 217–223. (doi:10.1111/j.2041-210X.2011.00169.x)

5. Ellinghaus D, Kurtz S, Willhoeft U. 2008 LTRharvest, an efficient and flexible software for de novo detection of LTR retrotransposons. *BMC Bioinformatics* 9, 18. (doi:10.1186/1471-2105-9-18)

6. Llorens C *et al.* 2011 The Gypsy Database (GyDB) of mobile genetic elements: release 2.0. *Nucleic Acids Res* 39, D70–D74. (doi:10.1093/nar/gkq1061)

7. Panchy N, Lehti-Shiu M, Shiu S-H. 2016 Evolution of Gene Duplication in Plants. *Plant Physiol* 171, 2294–2316. (doi:10.1104/pp.16.00523)

8. Wang Y *et al.* 2012 MCScanX: a toolkit for detection and evolutionary analysis of gene synteny and collinearity. *Nucleic Acids Res* 40, e49. (doi:10.1093/nar/gkr1293)

9. Xu L *et al.* 2019 OrthoVenn2: a web server for whole-genome comparison and annotation of orthologous clusters across multiple species. *Nucleic Acids Res* 47, W52–W58. (doi:10.1093/nar/gkz333)

10. Iwasaki M *et al.* 2021 Identification of the sex-determining factor in the liverwort Marchantia polymorpha reveals unique evolution of sex chromosomes in a haploid system. *Current Biology* 31, 5522-5532.e7. (doi:10.1016/j.cub.2021.10.023)

11. Leebens-Mack JH *et al.* 2019 One thousand plant transcriptomes and the phylogenomics of green plants. *Nature* 574, 679–685. (doi:10.1038/s41586-019-1693-2)

12. Dong S *et al.* 2019 The Amount of RNA Editing Sites in Liverwort Organellar Genes Is Correlated with GC Content and Nuclear PPR Protein Diversity. *Genome Biology and Evolution* 11, 3233–3239. (doi:10.1093/gbe/evz232)
